# Genome-wide DNA methylation profiles distinguish silent from non-silent ACTH adenomas

**DOI:** 10.1007/s00401-020-02149-3

**Published:** 2020-03-17

**Authors:** Franz L. Ricklefs, Krystian D. Fita, Roman Rotermund, Andras Piffko, Simone Schmid, David Capper, Rolf Buslei, Michael Buchfelder, Till Burkhardt, Jakob Matschke, Katrin Lamszus, Manfred Westphal, Ulrich Schüller, Jörg Flitsch

**Affiliations:** 1 Department of Neurosurgery, University Medical Center Hamburg-Eppendorf, Hamburg, Germany; 2 Department of Neuropathology, Charité University Medical Center Berlin, Berlin, Germany; 3 Institute of Pathology, Sozialstiftung Bamberg, Bamberg, Germany; 4 Department of Neurosurgery, University of Erlangen-Nürnberg, Erlangen, Germany; 5 Department of Neurosurgery, Friedrich-Ebert-Hospital, Neumünster, Germany; 6 Institute of Neuropathology, University Medical Center Hamburg-Eppendorf, Hamburg, Germany; 7 Department of Pediatric Hematology and Oncology, University Medical Center Hamburg-Eppendorf, Hamburg, Germany; 8 Research Institute Children’s Cancer Center Hamburg, Hamburg, Germany

Corticotroph adenomas express adrenocorticotrophic hormone (ACTH) and may result in Cushing’s disease (CD), if associated with measurable or elevated blood ACTH and cortisol levels. Silent ACTH adenomas (SCA) that express ACTH, but do not cause hypercortisolism might exhibit a more aggressive course and are currently classified as “aggressive pituitary gland tumors” according to the WHO classification [5]. However, SCA and CD may only be distinguished by extensive endocrinological testing, which often is ambiguous and may include subclinical CD.

We analyzed tumor samples from patients, who underwent transsphenoidal resection of histologically proven ACTH adenomas (Fig. 1a) to detect DNA methylation-based subgroups that may predict the patients’ clinical phenotype. Twenty-three patients were clinically silent (SCA), whereas fourty-nine patients had an endocrinological proven CD. In general, SCA patients had significantly larger tumors as measured by MRI (Fig. 1b). Other details of patients and tumors are described in Online Supplementary Table 1.

**Fig.1:**
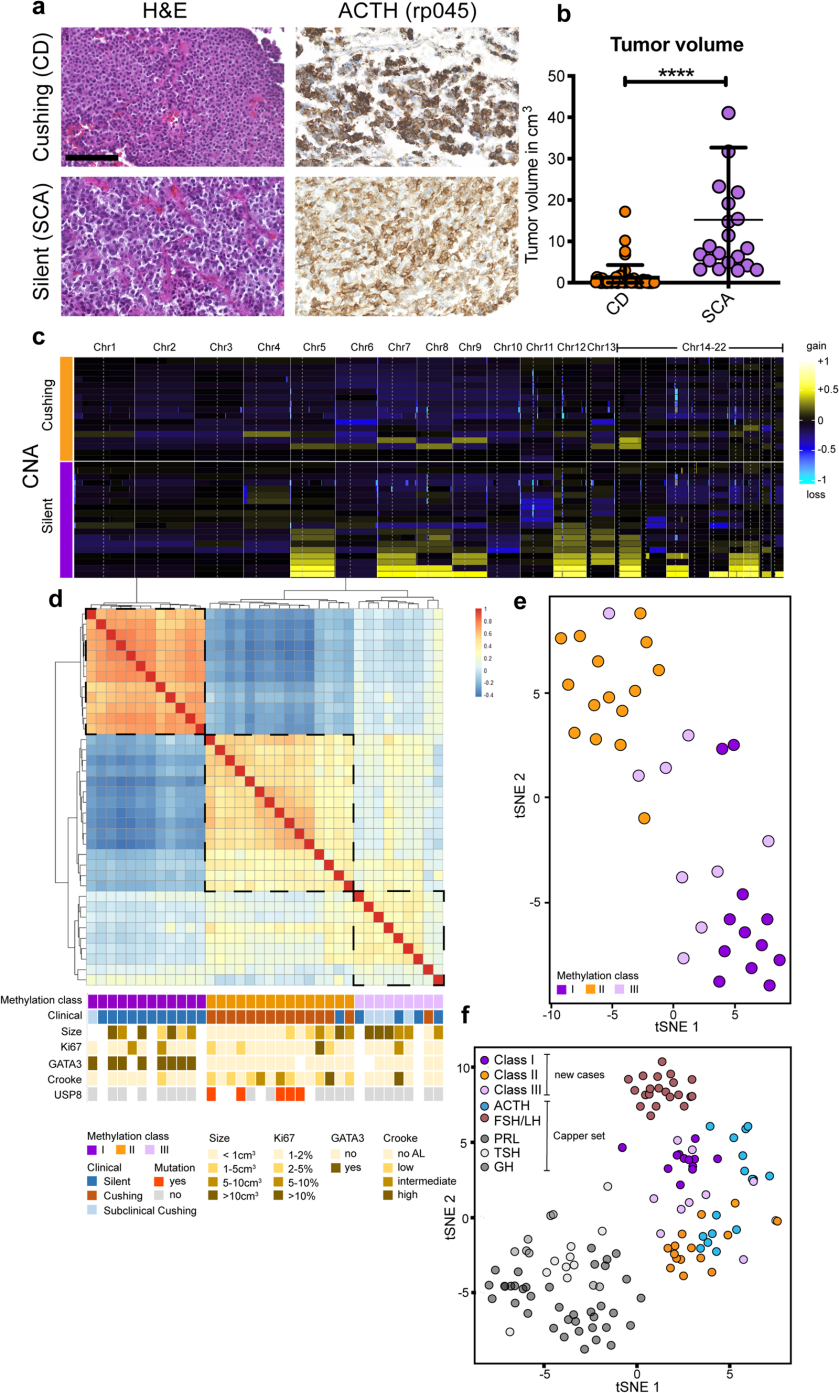
Genome-wide DNA methylation profiles split ACTH adenomas into clinically relevant subgroups. **a** Corticotroph adenomas causing either Cushing’s disease (CD) or being clinically silent (SCA) show strong expression of adrenocorticotrophic hormone (ACTH). Scale bar: 100 µm. **b** SCA patients have larger tumors in comparison to CD as measured by MRI (*p* < 0.0001). **c** Cumulative copy number alterations (CNA) inferred from the DNA methylation data. Healthy pituitary gland samples derived from the publicly accessible Capper set [2] were used as controls. **d** Heat map using pairwise Pearson correlation coefficients of the 10,000 most variable CpG features across all 36 samples reveals two distinct CD and SCA clusters and a third group with a clinically heterogeneous picture. No AL = no anterior lobe. **e***T*-Distributed Stochastic Neighbor Embedding (*t*-SNE) of all methylation probes indicates that SCA and CDs are distinguishable by their epigenetic profiles. **f***T*-SNE analysis with all 36 ACTH adenomas merged with the CNS tumor reference cohort from Capper et al. (2018).

By analyses of 850k or 450k Illumina arrays, we compared genome-wide DNA methylation profiles of 36 cases (CD:15 SCA:21). Copy number profiling inferred from DNA methylation data revealed cytogenetic aberration across all analyzed samples. SCA had a predominance for amplification of chromosomes 5, 7–9, 12–14, 16 and 19–20, yet only in 30–50% of cases (Fig. 1c).

Regarding DNA methylation, unsupervised hierarchical clustering of all samples using pairwise Pearson correlation coefficients distinguished CD and SCA as two separate clusters, while a third group was heterogeneous regarding its clinical manifestation (Fig. 1d). *USP8* mutations were only found in CD, but GATA3 expression exclusively appeared in SCA cases. *T*-distributed Stochastic Neighbor Embedding (*t*-SNE) further supported that SCA and CDs are distinguishable by their epigenetic profiles (Fig. 1e), which is specifically true for *POMC* regulatory gene regions (online supplementary Figure 1). Besides one case of CD, 4/8 non-CD cases within the heterogeneous third group had subclinical signs of CD, yet not fulfilling the required criteria to be diagnosed with CD [1]. Merging of our samples with the CNS tumor reference cohort from Capper et al. 2018 [2] reveals that about half of the reference cases cluster to our cases known as CD, whereas the remaining cases group together with SCA cases (Fig. 1f). Furthermore, gonadotroph adenomas show a spatial relationship with silent ACTH adenomas, which has also been suggested by Neou et al., who recently generated a pangenomic classification of pituitary tumors [6]. Since the DNA methylation imprint of a tumor cell is thought to occur at an early time point during tumorigenesis and to remain stable during progression [3], our data also imply a distinct tumor pathogenesis of CD and SCA adenomas with a potential “intermediate state” that clinically presents as a subclinical CD, but was as yet not separable neuropathologically. Indeed, our data suggest that genome-wide DNA methylation profiles allow subgrouping of SCA and CD adenomas that might not be achievable by standard endocrinological testing or histopathology.

## Electronic supplementary material

Below is the link to the electronic supplementary material.

Supplementary file1 (TIF 15419 kb)

Supplementary file2 (DOCX 50 kb)
