# Supplementary figures and images for: Genome-wide DNA methylation profiles distinguish silent from non-silent ACTH adenomas
Source: Acta Neuropathol. 2020 Mar 17;140(1):95–7. doi: 10.1007/s00401-020-02149-3 (PMC7299923; doi:10.1007/s00401-020-02149-3)

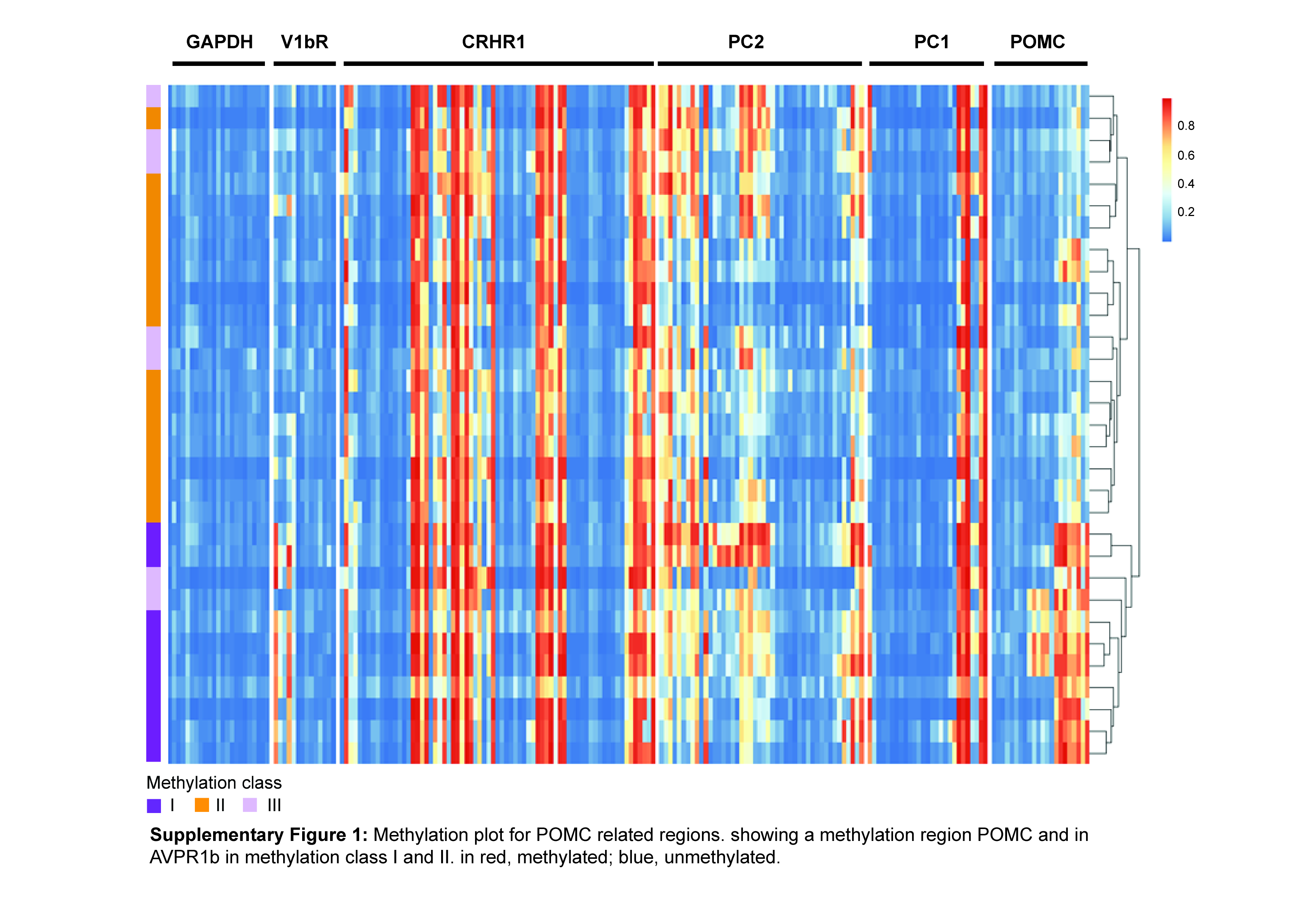

Supplement: Supplementary file 1 — Supplementary file1 (TIF 15419 kb) [file 401_2020_2149_MOESM1_ESM.tif]
