# Supplementary material for: Genome-wide DNA methylation profiles distinguish silent from non-silent ACTH adenomas
Source: Acta Neuropathol. 2020 Mar 17;140(1):95–7. doi: 10.1007/s00401-020-02149-3 (PMC7299923; doi:10.1007/s00401-020-02149-3)
Supplement: Supplementary file 2 — Supplementary file2 (DOCX 50 kb) [file 401_2020_2149_MOESM2_ESM.docx]

| Silent cortocotroph Adenoma | | | |  |  |  |  |  |  |  |  |  |  |  |  |  |  |  |
| --- | --- | --- | --- | --- | --- | --- | --- | --- | --- | --- | --- | --- | --- | --- | --- | --- | --- | --- |
| Patient | | Tumor volume | Operation | | ACTH | | | TSH | PRL | LH | FSH | GH | SF1 | GATA3 | Ki67 | Methylation (EPIC Array) | | |
|  |  |  |  |  | Granularity | Expression | |  |  |  |  |  |  |  |  |  |  |  |
| Age | Gender | CCM | Complete | Dekompression | 1=densely | Intensity | % positive | Intensity (0=none1=weak;2=mild;3=strong) | | | | | | 0=no | % positive | ID | Methylation | Methylation |
|  |  |  |  |  | 2=sparsely | 1=weak;2=mild;3=strong | cells |  |  |  |  |  |  | 1=yes | cells | Methylation | class | Score ACTH |
| 66 | m | 6.859 | 1 | 0 | na | 3 | 25 | 0 | 0 | 0 | 0 | 0 | 0 | 1 | 1-2 | silent 1 | I | na |
| 61 | f | 11.4264 | 1 | 0 | 2 | 2 | 100 | 0 | 3 | 0 | 0 | 0 | 0 | 0 | 1-2 | silent 2 | II | 0.99 |
| 48 | m | 3.264 | 1 | 0 | 1 | 1,2,3 | 30 | 0 | 0 | 0 | 0 | 0 | 0 | 1 | 1-2 | silent 3 | I | 0.96 |
| 76 | m | 7.1442 | 0 | 1 | 2 | 2 | 100 | 0 | 0 | 0 | 0 | 0 | 0 | 1 | 1-2 | silent 4 | I | 0.91 |
| 70 | f | 4.186 | 1 | 0 | 2 | 3 | 10 | 0 | 0 | 0 | 0 | 0 | 0 | 1 | 5 | silent 5 | I | 0.95 |
| 43 | m | 6.3 | na | na | na | 3 | 100 | 0 | 0 | 0 | 0 | 0 | 0 | 0 | 5 | silent 6 | III | 0.99 |
| 73 | m | 14.784 | 0 | 1 | 2 | 2 | 80 | 0 | 0 | 0 | 0 | 0 | 0 | 1 | 1-2 | silent 7 | I | 0.98 |
| 49 | m | 76.912 | 0 | 1 | 2 | 2+3 | 30 | 0 | 0 | 0 | 0 | 0 | na | na | 1-2 | silent 8 | III | 0.99 |
| 65 | m | na | 1 | 0 | 2 | 3 | 100 | 0 | 1 | 0 | 0 | 0 | na | na | 7 | silent 9 | I | 0.94 |
| 67 | m | na | 1 | 0 | 2 | 3 | 100 | 0 | 0 | 0 | 0 | 0 | 0 | 1 | 1-2 | silent 10 | I | 0.35 |
| 61 | f | 5.39028 | 1 | 0 | na | 3 | 5 | 0 | 0 | 0 | 0 | 0 | 0 | 0 | 1-2 | silent 11 | III | 0.99 |
| 63 | m | 31.7492 | 0 | 1 | 1 | 3 | 90 | 0 | 0 | 0 | 0 | 0 | 0 | 0 | 1-2 | silent 12 | III | 0.44 |
| 15 | m | 8.8587 | 1 | 0 | 2 | 2 | 90 | 0 | 0 | 0 | 0 | 0 | 0 | 0 | 1-2 | silent 13 | III | 0.99 |
| 52 | m | 15.454296 | 0 | 1 | 2 | 3 | 80 | 0 | 0 | 0 | 0 | 0 | 0 | 1 | 1-2 | silent 14 | I | na |
| 47 | m | 23.25 | 0 | 1 | 1 | 2 | 100 | 0 | 0 | 0 | 0 | 0 | 0 | 0 | 1-2 | silent 15 | III | 0.83 |
| 77 | m | 19.152 | 1 | 0 | 2 | 3 | 30 | 0 | 0 | 0 | 0 | 0 | 0 | 1 | 1-2 | silent 16 | I | 0.95 |
| 60 | m | 41.06223 | 0 | 1 | 2 | 3 | 70 | 0 | 0 | 0 | 0 | 0 |  |  |  |  |  |  |
| 70 | m | 2.943424 | 1 | 0 | 2 | 2 | 25 | 0 | 0 | 0 | 0 | 0 |  |  |  |  |  |  |
| 43 | f | 3.168 | 1 | 0 | 1 | 3 | 75 | 0 | 0 | 0 | 0 | 0 |  |  |  |  |  |  |
| 59 | m | 21.84 | 0 | 1 | 2 | 2 | 10 | 0 | 0 | 0 | 0 | 0 |  |  |  |  |  |  |
| 52 | m | 3.12 | 1 | 0 | 2 | 1 | 60 | 0 | 0 | 0 | 0 | 0 |  |  |  |  |  |  |
| 40 | f | 8.3538 | 1 | 0 | 2 | 2 | 80 | 0 | 0 | 0 | 0 | 0 |  |  |  |  |  |  |
| 53 | m | 4.913 | 1 | 0 | 2 | 2 | na | 0 | 0 | 0 | 0 | 0 |  |  |  |  |  |  |
|  |  |  |  |  |  |  |  |  |  |  |  |  |  |  |  |  |  |  |
| M. Cushing | | |  |  |  |  |  |  |  |  |  |  |  |  |  |  |  |  |
| 44 | f | 0.125 | 1 | 0 | 1 | 2 | 100 | 0 | 0 | 0 | 0 | 0 | 0 | 0 | 1-2 | Cushing1 | II | 0.99 |
| 19 | f | 0.125 | 1 | 0 | 1 | 3 | 100 | 0 | 0 | 0 | 0 | 0 | 0 | 0 | 5 | Cushing2 | II | 0.99 |
| 60 | f | 1 | 0 | 1 | 1 | 3 | > 75 | 0 | 0 | 0 | 0 | 0 | 0 | 0 | 1-2 | Cushing3 | III | 0.99 |
| 28 | f | 0.125 | 1 | 0 | 2 | 3 | 100 | 0 | 0 | 0 | 0 | 0 | 0 | 0 | 4 | Cushing4 | II | 0.98 |
| 24 | m | 1.053 | 1 | 0 | na | 3 | 100 | 0 | 0 | 0 | 0 | 0 | 0 | 0 | 4 | Cushing5 | II | 0.99 |
| 54 | f | 0.12 | 1 | 0 | 1 | 3 | 100 | 0 | 0 | 0 | 0 | 0 | 0 | 0 | 1-2 | Cushing6 | II | 0.99 |
| 56 | m | 6.84 | 0 | 1 | 1 | 3 | 100 | 0 | 0 | 0 | 0 | 0 | 0 | 0 | 1-2 | Cushing7 | II | 0.96 |
| 45 | f | 0.12 | 1 | 0 | 1 | 3 | 100 | 0 | 0 | 0 | 0 | 0 | 0 | 0 | 1-2 | Cushing8 | II | 0.98 |
| 46 | f | 1.368864 | 0 | 1 | 2 | 3 | 100 | 0 | 0 | 0 | 0 | 0 | 0 | 0 | 1-2 | Cushing9 | II | 0.96 |
| 37 | f | 1 | 1 | 0 | 1 | 2+3 | 50 | 0 | 0 | 0 | 0 | 0 | 0 | 0 | 3 | Cushing10 | II | na |
| 57 | f | 0 | 1 | 0 | 1 | 3 | > 90 | 0 | 0 | 0 | 0 | 0 | 0 | 0 | 1-2 | Cushing11 | II | 0.99 |
| 40 | f | 2.942784 | 1 | 0 | 2 | 3 | 75 | 0 | 0 | 0 | 0 | 0 | 0 | 0 | 1-2 | Cushing12 | II | 0.99 |
| 52 | f | 0.287631 | 1 | 0 | 1 | 3 | 90 | 0 | 0 | 0 | 0 | 0 | 0 | 0 | 1-2 | Cushing13 | II | 0.99 |
| 53 | m | 7.56 | 1 | 0 | 1 | 3 | >90 | 0 | 0 | 0 | 0 | 0 | 0 | 0 | 15 | Cushing14 | II | 0.98 |
| 53 | f | 0.729 | 1 | 0 | 1 | 3 | 100 | 0 | 0 | 0 | 0 | 0 | na | na | 1-2 | Cushing15 | II | 0.99 |
| 50 | f | 0.048 | 1 | 0 | 1 | 3 | > 90 | 0 | 0 | 0 | 0 | 0 |  |  |  |  |  |  |
| 17 | m | 0.018 | 1 | 0 | 1 | 3 | > 75 | 0 | 0 | 0 | 0 | 0 |  |  |  |  |  |  |
| 43 | f | 0.064 | 1 | 0 | 1 | 3 | > 75 | 0 | 0 | 0 | 0 | 0 |  |  |  |  |  |  |
| 63 | f | 0.176824 | 1 | 0 | 1 | 3 | 100 | 0 | 0 | 0 | 0 | 0 |  |  |  |  |  |  |
| 56 | f | 0.11088 | 1 | 0 | 1 | 3 | 100 | 0 | 0 | 0 | 0 | 0 |  |  |  |  |  |  |
| 25 | f | 0.036 | 1 | 0 | 1 | 3 | 90 | 0 | 0 | 0 | 0 | 0 |  |  |  |  |  |  |
| 56 | f | 17.18472 | 1 | 0 | 2 | 2+3 | 60 | 0 | 0 | 3 | 0 | 0 |  |  |  |  |  |  |
| 39 | f | 0.16461 | 1 | 0 | 1 | 3 | 80 | 0 | 0 | 0 | 0 | 0 |  |  |  |  |  |  |
| 30 | m | 0.024 | 1 | 0 | 2 | 3 | 100 | 0 | 0 | 0 | 0 | 0 |  |  |  |  |  |  |
| 78 | m | 0.727904 | 0 | 1 | 2 | 3 | > 90 | 0 | 0 | 0 | 0 | 0 |  |  |  |  |  |  |
| 43 | f | 0.59392 | 1 | 0 | 1 | 3 | > 90 | 0 | 3 | 0 | 0 | 0 |  |  |  |  |  |  |
| 9 | f | 0.064 | 1 | 0 | 2 | 3 | > 75 | 0 | 0 | 0 | 0 | 0 |  |  |  |  |  |  |
| 76 | f | 10.207296 | 0 | 1 | 2 | 3 | 15 | 0 | 0 | 0 | 0 | 0 |  |  |  |  |  |  |
| 60 | f | 0.21 | 1 | 0 | 1 | 3 | > 90 | 0 | 0 | 0 | 0 | 0 |  |  |  |  |  |  |
| 83 | m | 1.68 | 1 | 0 | na | 2 | 100 | 0 | 0 | 0 | 0 | 0 |  |  |  |  |  |  |
| 36 | m | 0.24 | 1 | 0 | 1 | 3 | 100 | 0 | 0 | 0 | 0 | 0 |  |  |  |  |  |  |
| 48 | f | 0.618772 | 1 | 0 | 2 | 3 | 75 | 0 | 0 | 0 | 0 | 0 |  |  |  |  |  |  |
| 41 | m | 1.342914 | 1 | 0 | 2 | 3 | 95 | 0 | 0 | 0 | 0 | 0 |  |  |  |  |  |  |
| 58 | m | 0.125 | 1 | 0 | 1 | 3 | 100 | 0 | 0 | 0 | 0 | 0 |  |  |  |  |  |  |
| 42 | f | 0.027 | 1 | 0 | 1 | 3 | 100 | 0 | 0 | 0 | 0 | 0 |  |  |  |  |  |  |
| 53 | f | 0.714816 | 1 | 0 | 2 | 3 | 80 | 0 | 0 | 0 | 0 | 0 |  |  |  |  |  |  |
| 37 | f | 0.027 | 0 | 1 | 1 | 3 | 100 | 0 | 0 | 0 | 0 | 0 |  |  |  |  |  |  |
| 53 | f | 0.414 | 1 | 0 | 1 | 3 | 100 | 0 | 0 | 0 | 0 | 0 |  |  |  |  |  |  |
| 33 | f | 0.079695 | 1 | 0 | 1 | 3 | 90 | 0 | 0 | 0 | 0 | 0 |  |  |  |  |  |  |
| 48 | m | 0.002 | 1 | 0 | 1 | 3 | 90 | 0 | 0 | 0 | 0 | 0 |  |  |  |  |  |  |
| 39 | f | 0 | 1 | 0 | 1 | 3 | 90 | 0 | 0 | 0 | 0 | 0 |  |  |  |  |  |  |
| 29 | m | 0 | 1 | 0 | 1 | 3 | 90 | 0 | 0 | 0 | 0 | 0 |  |  |  |  |  |  |
| 46 | f | 0.12 | 1 | 0 | 1 | 2 | 100 | 0 | 0 | 0 | 0 | 0 |  |  |  |  |  |  |
| 64 | f | 0.100096 | 1 | 0 | 1 | 3 | 100 | 0 | 0 | 0 | 0 | 0 |  |  |  |  |  |  |
| 51 | f | 0.217 | 1 | 0 | 1 | 3 | 100 | 0 | 0 | 0 | 0 | 0 |  |  |  |  |  |  |
| 45 | f | 0.59168 | 1 | 0 | 2 | 2+3 | 70 | 0 | 0 | 0 | 0 | 0 |  |  |  |  |  |  |
| 31 | m | 0.004 | 1 | 0 | na | 3 | 100 | 0 | 0 | 0 | 0 | 0 |  |  |  |  |  |  |
| 35 | m | 0 | 1 | 0 | na | 3 | 100 | 0 | 0 | 0 | 0 | 0 |  |  |  |  |  |  |
| 62 | f | 0.18 | 1 | 0 | 1 | 3 | 100 | 0 | 0 | 0 | 0 | 0 |  |  |  |  |  |  |
